# Reduced Face Aftereffects in Autism Are Not Due to Poor Attention

**DOI:** 10.1371/journal.pone.0081353

**Published:** 2013-11-29

**Authors:** Louise Ewing, Katie Leach, Elizabeth Pellicano, Linda Jeffery, Gillian Rhodes

**Affiliations:** 1 Australian Research Council Centre of Excellence in Cognition and its Disorders, School of Psychology, University of Western Australia, Perth, Australia; 2 Centre for Research in Autism and Education (CRAE), Institute of Education, University of London, London, United Kingdom; Istituto di Neuroscienze, Italy

## Abstract

This study aimed to determine why face identity aftereffects are diminished in children with autism, relative to typical children. To address the possibility that reduced face aftereffects might reflect reduced attention to adapting stimuli, we investigated the consequence of controlling attention to adapting faces during a face identity aftereffect task in children with autism and typical children. We also included a size-change between adaptation and test stimuli to determine whether the reduced aftereffects reflect atypical adaptation to low- or higher-level stimulus properties. Results indicated that when attention was controlled and directed towards adapting stimuli, face identity aftereffects in children with autism were significantly reduced relative to typical children. This finding challenges the notion that atypicalities in the quality and/or quantity of children’s attention during adaptation might account for group differences previously observed in this paradigm. Additionally, evidence of diminished face identity aftereffects despite a stimulus size change supports an adaptive processing atypicality in autism that extends beyond low-level, retinotopically coded stimulus properties. These findings support the notion that diminished face aftereffects in autism reflect atypicalities in adaptive norm-based coding, which could also contribute to face processing difficulties in this group.

## Introduction

Adaptive coding, or the updating of perception through experience, is widely thought to enhance processing across perceptual domains [1-3]. In face-processing, ongoing calibration of perception around a normative face representation may ensure that the limited range of neural responses are ‘tuned’ to prevailing inputs [2-4]. Such tuning would reduce the salience of common properties to favour the encoding of more novel or distinctive information and facilitate efficient face discrimination. 

Face aftereffects, i.e., shifts in perception that occur following exposure (adaptation) to faces, provide a potential index of adaptive coding [4]. In adults, these high-level aftereffects have been observed for every facial characteristic investigated, including identity [5], ethnicity [6], and expression [6]. Furthermore, recent developmental research reports similar findings with children [7-11], suggesting that adaptive face coding mechanisms mature and function early in typical development [12]. 

**Figure 1:**
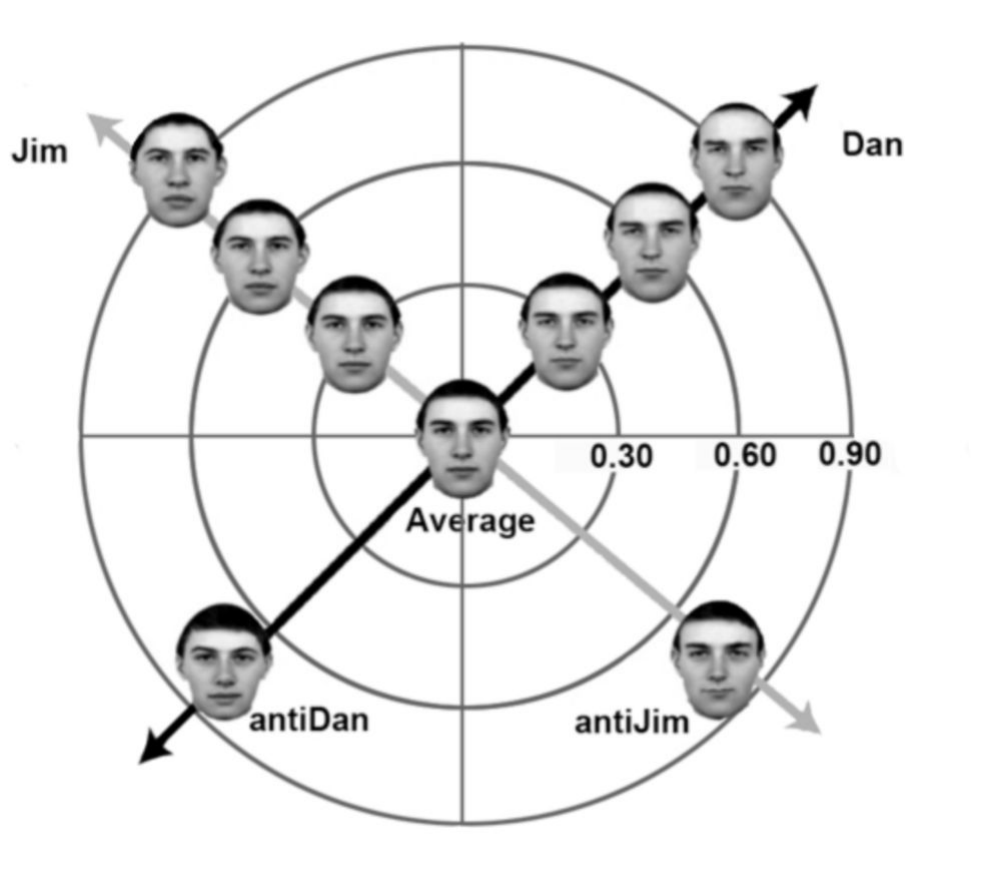
Simplified representation of face space, e.g., [42]. The average or norm face lies at the centre, and more distinctive faces lie towards the perimeter. Two example identity trajectories are shown, each extending from the original face (Dan or Jim, 90% identity strength), to the average face or norm (0% identity strength) through to the antiface which has complementary characteristics to the original face and negative identity strength values (e.g., -80%). In the face identity aftereffect, exposure to a face (e.g., Anti-Dan) shifts the average (norm) towards that face, making the opposite face (e.g., Dan) now appear further from the norm. This shift makes the original face (e.g., Dan) now appear more distinctive and easier to recognize at weaker identity strengths [5].

In contrast, there is growing evidence that face aftereffects are selectively diminished in children with autism – a clinical group widely observed to show face processing difficulties [13,14]. Adaptation to distorted faces (manipulated to appear expanded or contracted) and faces with left- and right-averted gaze results in perceptual shifts that are significantly reduced in children with autism relative to typical children of similar age and cognitive ability [15,16]. Similarly, adaptation to (anti)face identities results in a bias to recognize perceptually opposite identities, a face identity aftereffect, that is significantly smaller in children with autism relative to typical children [17], see Figure 1. Intriguingly, this diminished face identity aftereffect is also observed in the parents and siblings of children with autism [18], as well as neurotypical males with high levels of autism-like traits [19], which may indicate that diminished adaptive processes represent a neurocognitive endophenotype for autism.

It is important to consider, however, that atypical adaptive coding is only one of several potential explanations for reduced face aftereffects in autism. It is possible, for example, that these diminished face aftereffects could also reflect atypical attention. Attention has been shown to powerfully modulate the magnitude of perceptual adaptation in adults, with face aftereffects increasing with enhanced attention to adapting stimuli [20] and diminishing with reduced visual awareness of adapting stimuli [21-23] but see 24. In the studies reporting atypical face aftereffects in children with autism, participants were closely monitored to ensure that they fixated the stimuli presented on the screen. Nevertheless, given that diminished social interest and motivation to attend to faces is highly characteristic of autism [25-27] it is difficult to rule out subtle group differences in children’s attention to the adapting faces. Reduced attention to adapting face stimuli could therefore have plausibly contributed to diminished face aftereffects in children with autism, relative to typical children. 

The current study aimed to test directly whether diminished face aftereffects in autism persist when children’s attention to adapting stimuli is controlled, and directed towards these faces. To this end, we measured face identity aftereffects in children with and without autism under two attention conditions. In one condition, participants viewed adapting face stimuli passively (‘Standard’ condition) and in the other (‘Attention-control’ condition) they were required to concurrently detect brightness changes to the eyes or lips of adapting faces, as in [20]. Attention condition was kept as a between-participants factor to prevent potential interactions with order [20]. Persistence of a group difference when attention is controlled would strengthen the case for an adaptive coding atypicality in children with autism.

We also asked whether reduced face identity aftereffects in children with autism are driven by low- or higher-level adaptive coding atypicalities. Face aftereffects can reflect adaptation to lower-level stimulus features such as contrast, shape and tilt [4] as well as higher-level visual properties [28,29]. Recently, Ewing and colleagues presented evidence that diminished face distortion aftereffects constitute a high-level perceptual atypicality in autism [15]. Pellicano and colleagues [17] did not rule out a low-level origin for the reduced face identity aftereffects. Therefore the current study included a stimulus size change between adaptation and test, to reduce low-level retinotopic adaptation and ensure that observed atypical face aftereffects reflect a higher-level perceptual difference in the children with autism.

## Method

### Ethics Statement

The study was approved by the Human Research Ethics Committee at the University of Western Australia and all parents provided written consent prior to their child’s participation in the project. All children also gave verbal assent before taking part and some older children and adolescents also provided written consent.

### Participants

Twenty-one cognitively able children with autism (16 boys) aged 8 years 8 months to 16 years 0 months were recruited from local schools, community groups and the West Australian Register for Autism Spectrum Disorders (see Table 1). These children had received independent diagnoses of either Autistic Disorder (n=17) or Asperger’s Disorder (n=4) by a multidisciplinary team, see 30 for details, following DSM-IV criteria [31]. They also completed Modules 3 or 4 of the Autism Diagnostic Observation Schedule – Generic (ADOS-G) [32]. Six children scored below the algorithm cut-offs for autism spectrum disorder on this measure (3 in each attention condition), which indicated that their levels of *current* autistic symptomatology were not sufficient to meet ADOS-G criteria for autism. Nevertheless, all parents rated their child above the cut-off score of 15, indicative of clinically-significant levels of autistic symptomatology, on the Social Communication Questionnaire (SCQ) [33]. Two additional children with autism were also tested, but excluded prior to participant matching because their responses were poorly fitted by a cumulative Gaussian curve (see below). These children did not differ from the final sample with regards to their age (*p*=.10), verbal ability (VIQ, *p*=.64), non-verbal ability (NV-IQ, *p*=.84), or their autism symptomatology as measured by the ADOS-G (*p*=.10), the SCQ (*p*=.71), or the Social Responsiveness Scale (SRS) [34], p=.69].

**Table 1:** Mean (SD) for Chronological Age, Cognitive Ability, and ADOS-G scores in each attention condition.

	Standard Condition			Attention-control Condition		
	Mean (SD)			Mean (SD)		
	Typical (n=18)	Autism (n=12)		Typical (n=17)	Autism (n=9)	
Age (*months*)	136.7 (30.6)	151.8 (28.3)	*t(28) = 1.36, p = .18*	143.4 (30.7)	148.4 (27.9)	*t(24) = .41, p = .68*
Non-verbal IQ	101.7 (11.3)	98.2 (13.8)	*t(28) = -.76, p = .45*	103.1 (12.1)	100.9 (15.6)	*t(24) = -.40, p = .68*
Verbal IQ**^*a*^**	104.2 (11.4)	100.0 (12.6)	*t(28) =-.93, p = .35*	105.0 (10.0)	99.4 (15.6)	*t(24) = -1.11, p = .27*
SCQ**^*b*^**, **^*c*^**	2.5 (2.3)	26.8 (5.5)	*t(28) =16.96, p < .001*	3.3 (2.5)	23.7 (5.4)	*t(24) = 13.33, p < .001*
SRS**^*b*^**	14.2 (11.2)	100.3 (28.0)	*t(28) =11.79, p < .001*	18.6 (17.3)	103.0 (25.5)	*t(24) = 10.03, p < .001*
ADOS-G		10.3 (5.7)			7.0 (3.9)	

Notes. ***^a^*** Non-verbal and Verbal IQ were each measured with two subtests of the WISC-IV (Wechsler, 2003); NV-IQ = Matrix Reasoning and Picture Completion, V-IQ = Similarities and Vocabulary. ***^b^*** Higher scores on the SCQ (Lifetime form of the Social Communication Questionnaire; Rutter et al., 2003), ADOS-G (Autism Diagnostic Observation Schedule – Generic; Lord et al., 2000) and SRS (Social Responsiveness Scale; Constantino et al., 2000) indicate increased symptoms. SRS score reported = total raw score (max =195). ADOS-G score reported = Communication + Social Interaction algorithm total (cutoffs: autism = 10, autism spectrum = 7)

Thirty-five typically developing children also participated (27 boys). They were of similar chronological age (*p*=.20), non-verbal ability (*p*=.38) and verbal ability (*p*=.14) to our final sample of children with autism (Table 1). Relative to the children with autism, typical children had significantly lower scores on the SCQ (*p*< .001) and the SRS (*p*< .001).

Participants in each group were assigned to one of two attention conditions: Standard or Attention-control. This division of participants did not generate significant within-group differences in age, cognitive ability or autism symptomatology (2 subgroups of children with autism, all ts< 1.46, ps >.15; 2 subgroups of typical children, all ts< .98, ps >.33) (Table 1). 

### Stimuli

**Figure 2:**
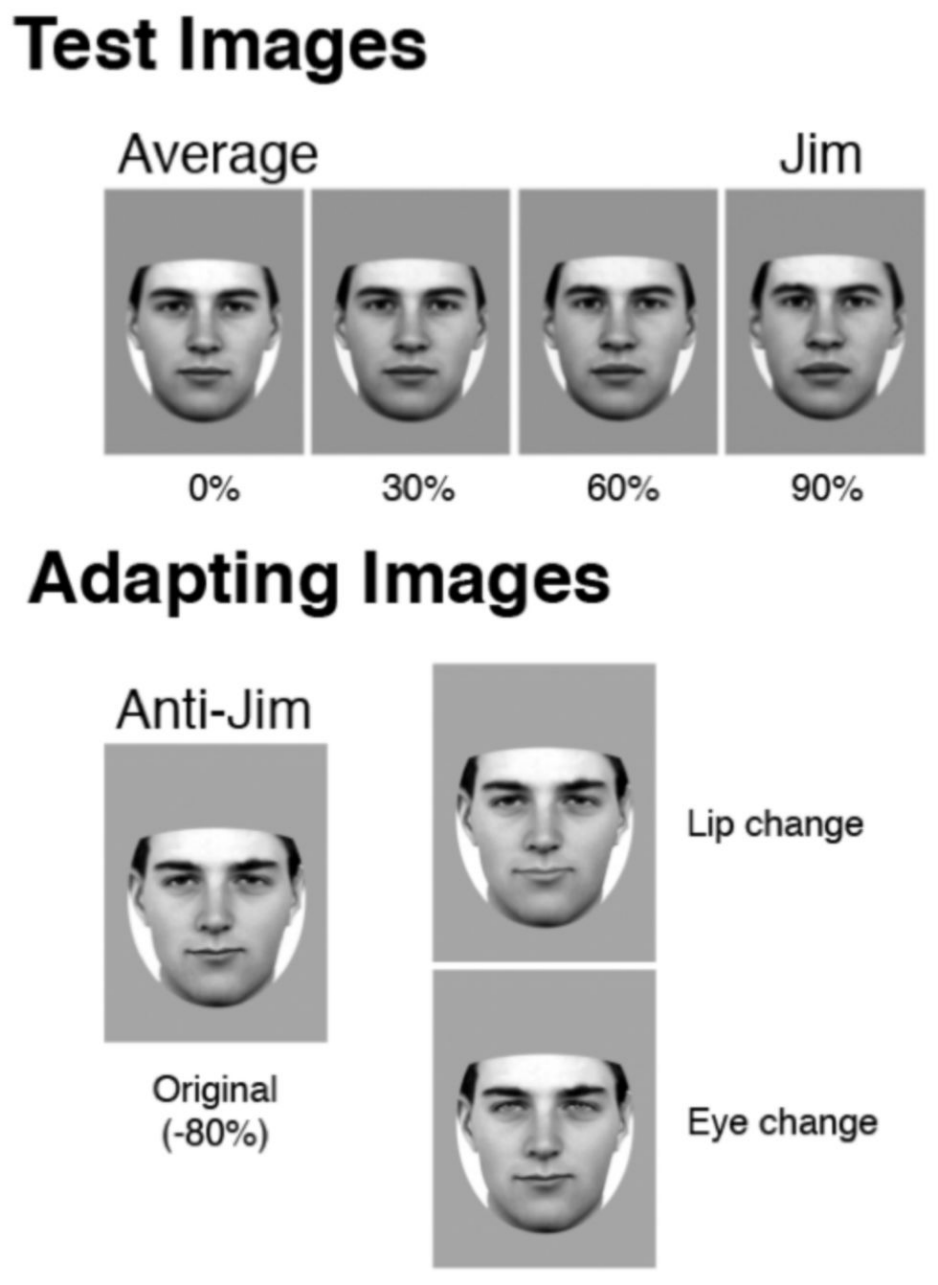
Sample test and adapting stimuli used in the Standard and Attention-control attention condition of the adaptation task (exemplars from the Dan and Jim identity pair are shown here). The “Lip change” and “Eye change” versions of the anti-face were used in the change detection task in the Control attention condition.

Four greyscale male faces (Dan, Jim, Ted, Rob) with neutral expressions and direct gaze were morphed with a 20-face male average face to make ‘reduced strength’ versions (30%, 40%, 60%, and 90%: test faces) and an antiface (-80%: adapting faces) for each identity, created by [35] see Figure 2. We also used Adobe Photoshop to create a version of each anti-face with lightened irises (40% increased brightness) and another version with lightened lips (20% increased brightness) for use in the Attention-control condition. Test images subtended an average visual angle of approximately 5.5° × 4.7°, and adapt-faces 6.3° × 6.4°, from a viewing distance of 57cm. 

### Procedure

The adaptation task, which took the form of a Robbers Game [17], was administered in a quiet room at home on a 15 inch Apple PowerBook Pro laptop computer using SuperLab Pro 1.75 software, as part of a larger battery of behavioural tasks. In the game, children viewed faces presented onscreen in order to determine whether a series of robbers (adapting faces) were apprehended by one crime fighting team or another (test faces). The crime fighting teams belonged to either ‘Dan and Jim’ or ‘Ted and Rob’, counter-balanced between participants. For ease of exposition only the Dan and Jim identity pair is described below. Participants completed either the Standard or Attention-control condition of this game. Critically, these conditions only differed during the adaptation phase of the game.

#### Training

During this phase children learned to identify the two target identities. Participants were first introduced to the “team captains” (e.g. Dan and Jim) at 100% identity strength on paper printouts. Then 20 images of these two faces were presented on screen in a pseudorandom order (first block of ten trials = unlimited exposure duration, second block = 400ms exposures) for children to identify via labelled key-press. Auditory performance feedback was provided (“boing” and “quack” sounds for correct and incorrect trials respectively). Success on 4 of the 5 final trials was required for them to progress to the next part of their training. One typical child repeated these blocks once. 

Participants were then trained also to recognize Dan and Jim’s team-mates: weaker strength versions of the two target identities. Dan and Jim were each shown four times at 40, 60, and 100% strengths four times in a pseudorandom order (first block of 12 trials = unlimited exposure duration, second block of 12 trials = 400ms exposures). Again, children responded with a labelled key-press, received auditory performance feedback, and were required to succeed on 4 of the 5 final trials to progress forward. One child with autism repeated these blocks once.

#### Discrimination

During this phase, we confirmed that participants could perform the Robbers Game by assessing their ability to discriminate between members of the two crime fighting teams (in the absence of adaptation). After briefly viewing the full and reduced identity strength versions of the two target identities on screen, children completed 48 discrimination trials (Dan and Jim at 0, 30, 60, and 90% strength x 6, divided into 3 equal blocks). On each trial, a space-bar press initiated the presentation of a face for 400ms, which was to be identified as a member of either Dan or Jim’s team with a labelled key-press. No performance feedback was provided. Trial order was pseudo-randomised, with the constraint that each test face was presented twice per block.

#### Adaptation: Standard condition

Attention was not controlled in this condition. Here, on each trial, the face of a robber (Anti-Dan or Anti-Jim) flashed four times (1000ms each), then a test face appeared for 400ms (Dan or Jim 0, 30, 60, 90%) and a response screen prompted the child to indicate “Which team caught the robber?” with a labelled key-press. Performance feedback was not provided. Both adapt faces were shown with both test faces at all four identity strengths, 6 times. The resulting 96 trials were divided into 6 equal blocks, in which every trial combination of adapt face and test face appeared once every two blocks. Before commencing each block, participants were briefly reminded of the faces of the two crime fighting teams. At the end of each block participants received encouraging feedback on their performance and viewed pre-generated statistics on the robbers that had been successfully caught. 

The trial order in this phase was pseudo-randomized with the constraint that the same antiface could not appear on more than two consecutive trials, to prevent a build-up of adaptation to one face. Additionally, the same test identity could not appear on more than three consecutive trials, to avoid perseverative responses in children. Two different trial orders were generated, and these were counterbalanced between participants. 

#### Adaptation: Attention-control condition

We controlled children’s attention during the adaptation phase in this condition. All aspects of the procedure were the same as in the Standard condition, with the following exception. Children were told that the robbers were “extra sneaky” and tried to evade capture by changing the shade of their eyes and lips. They therefore had to watch the robber’s face carefully, because on the second or third “flash” exposure of his face (equally likely) he had lightened eyes or lips (equally likely). They were to call out “Eyes” or “Lips” as soon as they noticed a change, in addition to indicating via key-press which team caught the robber at the end of the trial. The experimenter recorded the change-detection responses using the keyboard, to minimize possible interference between the eye/lip change-detection and identity judgement components of this task. In a further effort to increase attention during adaptation, children were also told that the top-scoring children on this task had been those that had, “looked at the robbers face for the whole time he flashed up”.

Prior to commencing this phase, children were shown examples of the eye and lip change stimuli on paper printouts and on screen, to ensure that they could correctly identify and report these changes. Every possible trial combination of adapt face, eye/lip change, and test face appeared once every two blocks.

## Results

We measured attention to the adapting faces in the Attention-control condition by calculating children’s performance accuracy (percentage correct) on the change detection task. Performance was almost perfect in both the typical children (*M*= 99.6, *SD*=0.8) and the children with autism (*M*=99.0, *SD*=2.0), confirming that participants attended closely to the adapting stimuli. 

**Figure 3:**
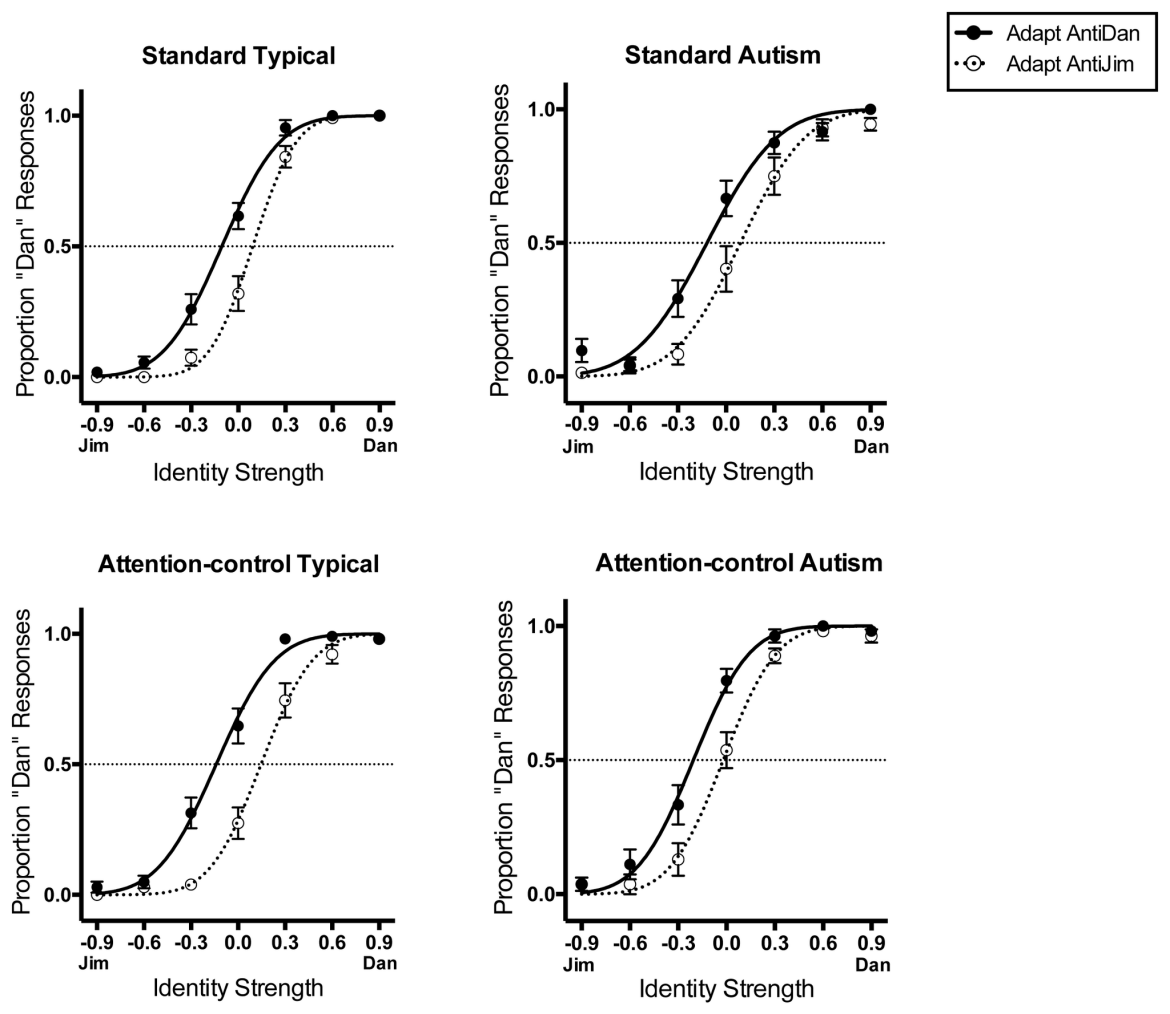
Cumulative Gaussians fitted to group data for in the Standard (left) and Attention-control condition (right) for typical children and children with autism. SE bars are shown. All aftereffects used in analysis were calculated from curves fitted to individual data.

Each child’s face identity aftereffect was measured as degree to which target faces were identified as being the identity (e.g., Dan) opposite to the adapting face (e.g., Anti-Dan). For example, targets should be identified as “Dan” more often after adapting to Anti-Dan, which biases perception toward Dan, than after adapting to Anti-Jim, which biases perception toward Jim. Each participant’s proportion of “Dan” responses was therefore plotted as a function of identity strength after adaptation to Anti-Dan and Anti-Jim (Note - “Dan”/“Anti-Dan” refers here to Dan or Ted, and “Jim”/“Anti-Jim” refers to Jim or Rob, depending on the identity pair assigned to the participant). These curves were fit with cumulative Gaussian functions (all *R* ^2^ > 0.6, see Figure 3 for group data). An aftereffect in the predicted direction is indicated by the adapt Anti-Dan curve being to the left of the adapt Anti-Jim curve. The mean of each function represents the Point of Subjective Equality (PSE), i.e., the identity strength perceived to be ambiguous. Our dependent variable, the face identity aftereffect, was the difference between each child’s adapt Anti-Dan and adapt Anti-Jim PSEs, e.g. adapt Anti-Jim PSE minus adapt Anti-Dan PSE, scored so that a positive difference indicated an aftereffect in the predicted direction. Comparing outcomes from identically structured trials like this, rather than from a baseline and adapt phase, e.g., [17], ensured that any perceptual and attentional processes associated with exposure to faces were equated across conditions.

Values more than two standard deviations from each group’s mean in each condition were replaced with that value (mean +/- 2SD). One typical child’s score was replaced in the Standard condition and one typical child’s score was replaced in the Attention-control condition. The resultant distributions were normal and Levene’s test did not support significant heterogeneity of variance (*p*=.09). Significant adaptation occurred in both groups and attention conditions, with planned t-tests indicating that aftereffects were significantly greater than zero (all ts > 4.64, all *p*s < .01). 

**Figure 4:**
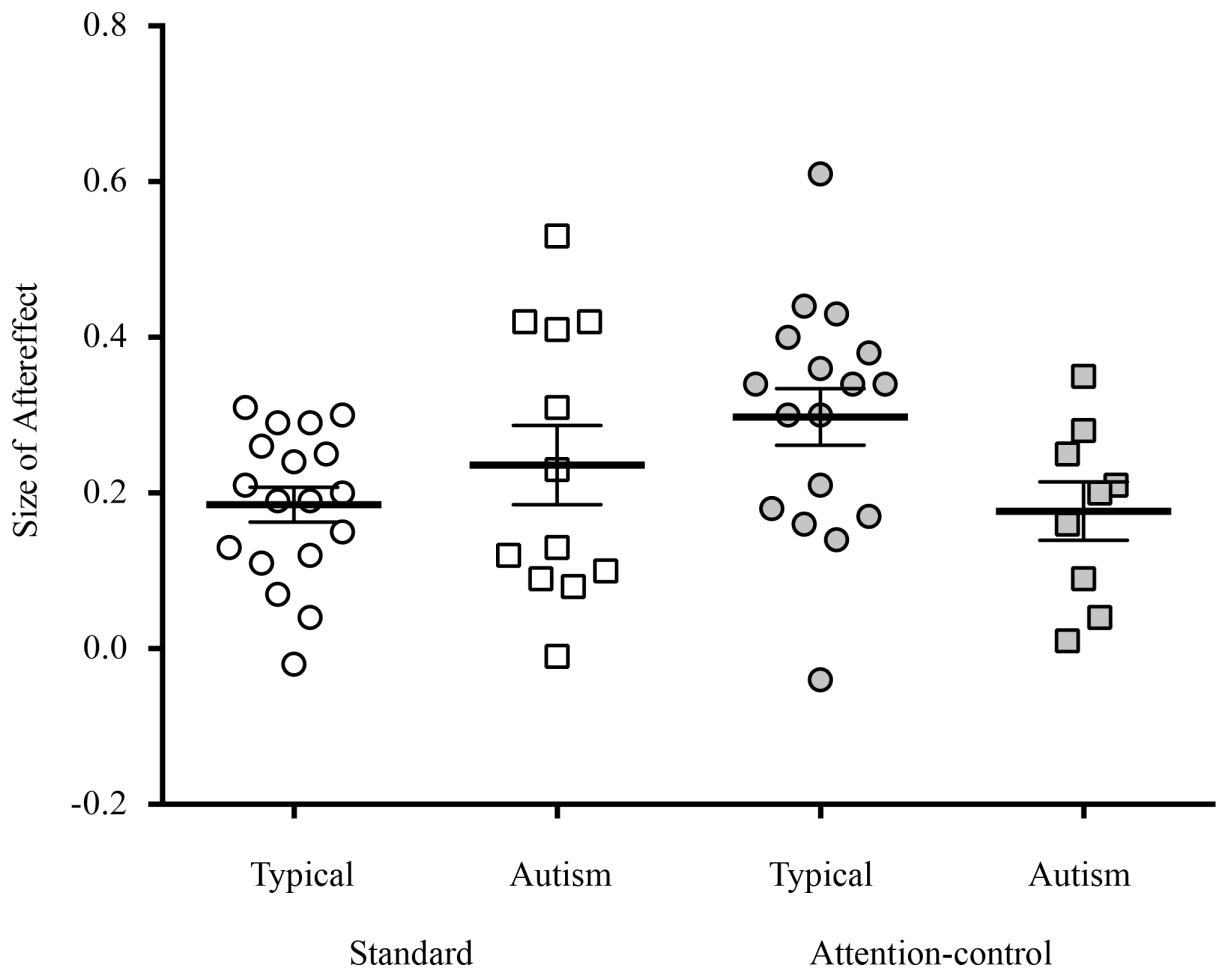
Aftereffects for participants in each group for the two attention conditions. The bold horizontal bars reflect the mean and standard error bars are shown.

A 2-way ANOVA examined the effects of participant group (typical, autism) and attention condition (Standard, Attention-control; between participants factor) on the magnitude of children’s face identity aftereffects (Figure 4). A preliminary 3-way ANOVA that also included identity pair (Dan and Jim, Ted and Rob) revealed that this additional variable produced no significant main effects or interactions (all *F*s < 1.39, all ps > .24) so these two conditions were collapsed. Unexpectedly, there was no significant effect of attention condition, *F*(1, 52) = 0.47, *p*=.49, η_p_^2^= .01 (Standard *M*=0.20, *SD*=0.13; Attention-control *M*=0.25, *SD*=0.14). There was also no significant effect of group, *F* (1, 52) = 0.90, *p* = .34, η_p_^2^ = .01 (typical *M*=0.24, *SD*= 0.13; autism *M*=0.21, *SD*= 0.15). 

There was, however, a significant interaction between group and attention condition, *F* (1, 52) = 5.25, *p* <.05, η_p_^2^ = .09. Planned comparisons, conducted separately for each attention condition, indicated that in the Attention-control condition, face identity aftereffects of children with autism (*M*=0.17, *SD*=0.11) were significantly reduced relative to those of typical children (*M*=0.29, *SD*=0.14), *t*(24) = 2.14, *p* <.05, d = 87. This evidence of diminished aftereffects in children with autism when their attention to adapting face stimuli was controlled, strongly suggests that atypical adaptation in this group reflects more than reduced attention to the adapting face stimuli. Unexpectedly, a similar effect was not observed in the Standard condition, *t*(28) = 1.01, *p* =.31, d = 0.38. In contrast to the findings of other face adaptation studies without attentional controls [15,17], we observed no significant difference in the magnitude of aftereffects in the autism group (*M*=0.23, *SD*=0.17) and the typical group (*M*=0.18, *SD*=0.09).

We also compared the magnitude of aftereffects in the two attention conditions for our participant groups. In the typical group, face aftereffects were larger in the Attention-control condition (*M*=0.29, *SD*=0.15), relative to the Standard condition (*M*=0.18, *SD*=0.09), *t*(33) = 2.66, *p* <.05, d = 0.90, as for adults. For the autism group, however, there was no significant difference in aftereffects in the Standard (*M*=.23, *SD*=.17) and Attention-control conditions (*M*=0.17, *SD*=0.11), *t*(19) = 0.89, *p*=.38, d=.40

Importantly, there was no significant difference between the groups in their ability to discriminate between the two well-learned target identities. A 2-way between subjects ANOVA was used to examine whether there were any effects of group (typical, autism) or attention condition (Standard, Attention-control) on identification precision, as measured as the mean slope (standard deviation) of Gaussian functions fit to each child’s discrimination data. This analysis revealed no significant main effects or interactions, *F*s < 1.6, ps > .20. Examination of post-adaptation identification precision (averaged across face identities) also confirmed the absence of a significant effect of attention condition on discrimination in typical children *t*(33) = .54, p=.58, and children with autism *t*(19) = .22, p=.81.

There was also no significant correlation between face aftereffects and autism symptoms in the clinical group as measured by combined total (Social Communication) scores on the ADOS-G (current symptoms, *r*(20) = .26, p = .25) or the SCQ (lifetime symptoms, *r*(20) = .25, *p* = .26).

## Discussion

This study demonstrates atypical adaptive face coding in autism when attention is controlled and low-level adaptation is minimized. This finding extends previous reports of diminished face identity aftereffects in children with autism [17] to show that reduced face aftereffects cannot be accounted for by reduced attention during adaptation. It also shows that these reduced aftereffects are not due solely to reduced adaptation to low-level stimulus properties. Ruling out these explanations for reduced face identity aftereffects in children with autism strengthens the case for atypical higher-level adaptive face coding in this group. 

Importantly, these adaptive coding atypicalities might contribute to face processing difficulties in children with autism, e.g., [13,14]. The mechanisms underlying these difficulties continue to be debated [27,36] and disrupted adaptive coding could provide an elegant explanation for problems in memory and discrimination. Efficient adaptive, norm-based coding has long been linked with face processing ability theoretically, see 4, and this association was recently confirmed empirically with typical adults. Individual differences in adaptive coding of identity, measured via face identity aftereffects, significantly predicted participants’ face recognition ability [37] see also 29. Clearly, the direct functional consequences of atypical adaptive coding in autism will be an interesting and important question for future research.

Interestingly, in the Standard-attention condition, face identity aftereffects were not smaller in children with autism than in typical children. This result contrasts with that of Pellicano and colleagues [17], who used a largely similar methodology, and a similar sized sample (n=14) with comparable symptom levels (*Mean SCQ score*=23.3). The reason for this difference is uncertain. Given that attention was not controlled in either case, differences in this variable may have played a role. However in our study and theirs, participants were closely monitored to ensure they were looking at the screen during the tasks, and the discrimination data does not suggest atypical attention, at least relative to the typical comparison groups, in either case. Another possibility is that face identity aftereffects are only reduced in autism when low-level adaptation is present. However, the reduced aftereffects seen in our Attention-control condition rule out this possibility. Instead, we suggest that the difference might be a sampling effect, i.e., differences between the autism groups in the two studies. This possibility is consistent with the phenotypic variability, including heterogeneity in face processing [38,39], which is characteristic of autism. 

In typical adults, the attention-control manipulation used here increases face identity aftereffects [20]. Our results suggest that the same is true for typical children. However, this was not the case for our autism groups. One possibility is that our attention instructions, to spot the eye/lip changes, may have had little effect on face processing in children with autism if they already adopt a feature-based processing style as has been proposed by [40,41]. We note, however, that adults with autism fail to show typical enhancements in face-selective brain responses with focused attention to faces [42,43], suggesting a potentially broader insensitivity to attention manipulations.

We used a between-participants design to avoid order effects associated with our attention manipulation [20]. This design, however, is not without its disadvantages, especially in light of both the heterogeneity of autism and individual differences in face processing in typical individuals. While there were no significant differences in age, cognitive ability or autism symptomatology between the children in our two autism groups, they might nevertheless have varied in other important ways, which may have affected our results. A within-subjects design could be useful in future studies, so long as task order effects can be avoided, see 20.

 In summary, our results indicate that diminished face identity aftereffects in children with autism are unlikely to reflect either reduced attention to faces or reduced adaptation to low-level stimulus features. Instead, they appear to reflect reduced adaptation to higher-level face attributes. 
